# Impaired Glutathione Synthesis in Neurodegeneration

**DOI:** 10.3390/ijms141021021

**Published:** 2013-10-18

**Authors:** Koji Aoyama, Toshio Nakaki

**Affiliations:** Department of Pharmacology, Teikyo University School of Medicine, 2-11-1 Kaga, Itabashi, Tokyo 173-8605, Japan; E-Mail: kaoyama@med.teikyo-u.ac.jp

**Keywords:** glutathione, cysteine transport, oxidative stress, neurodegeneration, EAAC1, GTRAP3-18

## Abstract

Glutathione (GSH) was discovered in yeast cells in 1888. Studies of GSH in mammalian cells before the 1980s focused exclusively on its function for the detoxication of xenobiotics or for drug metabolism in the liver, in which GSH is present at its highest concentration in the body. Increasing evidence has demonstrated other important roles of GSH in the brain, not only for the detoxication of xenobiotics but also for antioxidant defense and the regulation of intracellular redox homeostasis. GSH also regulates cell signaling, protein function, gene expression, and cell differentiation/proliferation in the brain. Clinically, inborn errors in GSH-related enzymes are very rare, but disorders of GSH metabolism are common in major neurodegenerative diseases showing GSH depletion and increased levels of oxidative stress in the brain. GSH depletion would precipitate oxidative damage in the brain, leading to neurodegenerative diseases. This review focuses on the significance of GSH function, the synthesis of GSH and its metabolism, and clinical disorders of GSH metabolism. A potential approach to increase brain GSH levels against neurodegeneration is also discussed.

## 1. Introduction

Glutathione (GSH) was discovered in yeast by two independent scientists over a century ago. In 1888, J. de Rey-Pailhade identified a substance from yeast cells, which he named “*philothione*” (from the Greek words meaning “love” and “sulfur”) because of its reactivity with sulfur to form hydrogen sulfide [1,2]. Subsequently, F.G. Hopkins reported this substance as a dipeptide containing glutamate and cysteine, and he named it “*glutathione*” [3], which is actually a tripeptide consisting of glutamate, cysteine, and glycine [4,5]. GSH is useful to weaken the strength of bread dough for baking, by its reaction with gluten in wheat. However, the significance of GSH function in living cells did not receive much attention until the 1970s, when a variety of studies on GSH-related biochemical reactions and its metabolism emerged. The studies of GSH since then have revealed that GSH is involved in pivotal cellular physiological processes, *i.e.*, antioxidant defense, detoxication of xenobiotics, intracellular redox homeostasis, cysteine carrier/storage, cell signaling, protein function, gene expression, and cell differentiation/proliferation. Thus, dysfunction of GSH metabolism can cause lethal cellular events. Early clinical studies of GSH metabolism focused solely on GSH-related enzyme dysfunction or the GSH-related metabolism of drugs and endogenous compounds in the liver. However, accumulating lines of evidence from recent studies indicate that disorder of GSH function is implicated in the etiology of some neurodegenerative diseases such as Alzheimer’s disease (AD), Parkinson’s disease (PD), amyotrophic lateral sclerosis (ALS), progressive supranuclear palsy (PSP), Huntington’s disease (HD), and multiple sclerosis (MS). This review focuses on the significance of GSH function, GSH synthesis and its metabolism, clinical disorders of GSH metabolism, and therapeutic strategies for the treatment of GSH-related neurodegenerative diseases.

## 2. Glutathione Synthesis

**Figure 1:**
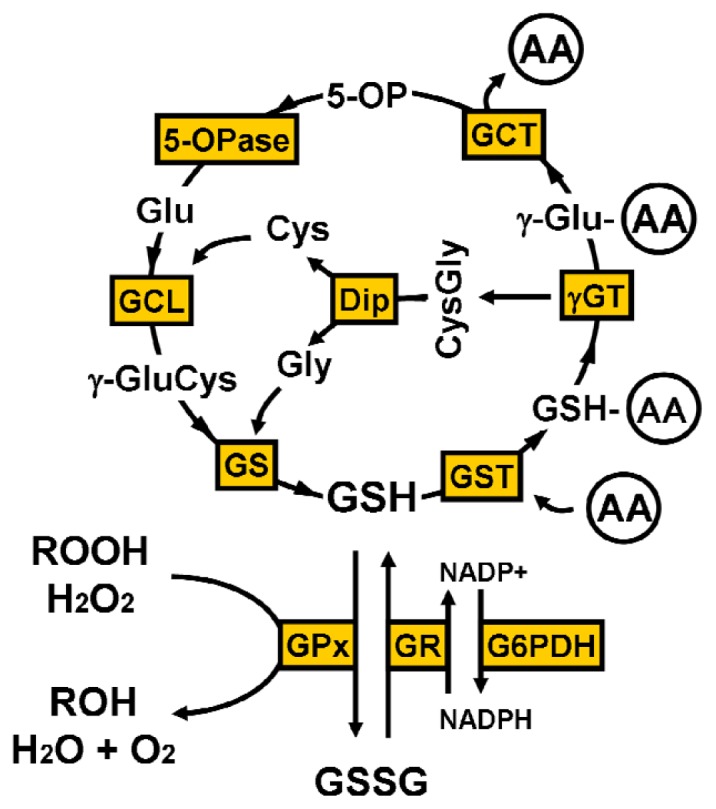
The γ-glutamyl cycle. AA, amino acids; Cys, cysteine; CysGly, cysteinylglycine; Dip, dipeptidase; GCL, γ-glutamylcysteine ligase; GCT, γ-glutamyl cyclotransferase; γGT, γ-glutamyl transpeptidase; γGluCys, γ-glutamylcysteine; Glu, glutamate; Gly, glycine; G6PDH, glucose-6-phosphate dehydrogenase; GPx, glutathione peroxidase; GR, glutathione reductase; GS, glutathione synthetase; GSH, glutathione; GSSG, glutathione disulfide; GST, glutathione-*S*-transferase; H_2_O_2_, hydrogen peroxide; NADPH, nicotinamide adenine dinucleotide phosphate; 5-OP, 5-oxoproline; 5-OPase, 5-oxoprolinase; ROH, alcohol; ROOH, hydroperoxide.

GSH is produced intracellularly from three amino acids—glutamate, cysteine and glycine—through two consecutive steps catalyzed by γ-glutamylcysteine ligase (GCL, also known as γ-glutamylcysteine synthetase) and GSH synthetase (GS) (Figure 1). GCL mediates the first step, which is the ATP-requiring reaction with glutamate and cysteine to form a dipeptide, γ-glutamylcysteine (γGluCys). The mammalian GCL is a heterodimer enzyme consisting of an approx. 73-kDa catalytic (heavy) subunit, GCLC, and an approx. 28-kDa modulatory (light) subunit, GCLM. GCLC, but not GCLM, has all the enzymatic activity and is subject to feedback inhibition by GSH [6]. GCLM has no enzymatic activity; however, the association of GCLM with GCLC decreases the *Km* value for glutamate and increases the *Ki* value for the feedback inhibition of GSH [7]. GS catalyzes the second step, another ATP-requiring reaction with γGluCys and glycine to form GSH, although much less is known about the precise mechanisms underlying the regulation of GS activity compared to those of GCL.

For GSH synthesis, the *Km* value of GCL for cysteine is ~0.15 mM, while that for glutamate is ~1.7 mM, and that of GS for glycine is ~0.8 mM [8]. The levels of intracellular glutamate (1–10 mM) [9] and glycine (2 mM in astrocytes, 10 mM in neurons) [10] are much higher than the *Km* values, whereas the intracellular cysteine level is around its *Km* value [8]. Although intracellular amino acid levels vary with tissues or cell types, cysteine concentrations in the brain are maintained at levels that are lower than those of glutamate or glycine [11] because of its neurotoxicity [12,13]. Therefore, the intracellular cysteine level is considered the rate-limiting factor for GSH synthesis in the brain.

## 3. Glutathione in the Brain

### 3.1. Distribution of GSH in the Brain

GSH serves approximately 95% of total non-protein thiol groups in living cells [14] and is ubiquitously distributed throughout the human body. However, GSH levels vary according to the organs (0.5–10 mM) [15,16]; the highest levels are found in the liver (5–10 mM), followed by the kidney, spleen, small intestine, brain, pancreas, lung, heart, and muscle [17]. The brain contains GSH at a varying concentration of approximately 2–3 mM [18] in different regions; it is highest in the cortex, followed by the cerebellum, hippocampus, and striatum, and lowest in the substantia nigra (SN) [19]. The GSH level in the cerebrospinal fluid (CSF) is much lower (~5 μM) than those of brain tissues [20,21]. The GSH levels also depend on cell types in the brain. Primarily, the GSH level is lower in neurons than in astrocytes [22]. Microglia contain higher GSH levels than neurons or astrocytes *in vitro* [23,24]. These findings suggest that the regulatory mechanisms of GSH metabolism/homeostasis are specific to the organs and cell types.

### 3.2. Thiol Source for the Brain

GSH is a non-toxic storage form of cysteine at levels 10–100 times higher than that of cysteine in mammalian tissues [18]. Approximately one-third to one-half of the total liver GSH serves as a cysteine reservoir that can be released into the blood as necessary [25,26]. However, the released GSH does not reach the brain directly because of the blood-brain barrier (BBB), which consists of a layer of capillary endothelial cells surrounded by astrocytes that functions as a selective barrier protecting the brain from xenobiotics in the blood. GSH can penetrate the BBB only poorly by the mechanism of passive diffusion, because of its hydrophilic property [27]. It is as yet unclear whether a direct GSH transport system exists at the BBB. Intravenously administered GSH is rapidly metabolized in blood [28,29]. Consistently, plasma GSH levels are much lower (2–20 μM) than those in the liver [16].

Cysteine is also unable to penetrate the BBB because of its lack of an acidic omega side chain, which facilitates the transport through the BBB [30]. In contrast, the disulfide form with two cysteines, called cystine, is transported from blood into the endothelial cells at the BBB via a cystine transporter, called system x_c_^−^, and is subsequently transported out of the endothelial cells into the CSF via the l-type amino acid transporter LAT1 at the BBB [31,32]. Favorably, cystine presents in the plasma at much higher concentrations (50–100 μM) than cysteine (10–25 μM), GSH, or other thiol derivatives [16,21,33]. These findings suggest that plasma cystine is the main thiol source of the brain [21].

### 3.3. Thiol Source for Glutathione Synthesis in the Brain

In the CSF, the cystine levels are relatively lower but the cysteine and GSH levels are higher compared to those in the blood [21]. These findings suggest a conversion of plasma cystine into other thiol derivatives in the central nervous system (CNS). Cystine is the primary source of GSH synthesis in astrocytes expressing system x_c_^−^, which is a sodium-independent cystine/glutamate antiporter composed of two subunits, xCT and 4F2hc [34], mainly present on glial cells [35,36]. Cystine imported into astrocytes is intracellularly reduced back to cysteine, which is used as a substrate for astroglial GSH synthesis. Astrocytes can also take up intact dipeptides, γGluCys and cysteinylglycine (CysGly), for GSH synthesis [37,38]. Astrocytes reserve high GSH contents at the concentration of approx. 8 mM [39] intracellularly, and they can export 10% of intracellular GSH within 1 h through multidrug resistance proteins (MRPs), which are members of the family of ATP-binding cassette transporters [40]. The released GSH is cleaved into γ-glutamyl moiety and CysGly by the reaction of an ectoenzyme on the glial plasma membrane, γ-glutamyl transpeptidase (γGT). CysGly is hydrolyzed by aminopeptidase *N* to cysteine and glycine, which are subsequently transported into neurons for GSH synthesis [41]. The released GSH may also react with intracellular cystine, which is transported from plasma, to form cysteine and cysteine-GSH. These metabolic interactions between astrocytes and neurons are essential as the source of cysteine or its precursor for neuronal GSH synthesis in the brain [42].

## 4. Glutathione Function

### 4.1. γ-Glutamyl Cycle

Reactive oxygen species (ROS) are produced during redox metabolism in cells. A large part (~90%) of ROS production is attributed to mitochondria. Initial observations indicated that approx. 2% of the total oxygen consumption is diverted to generate ROS [43], while more recent studies indicated that the basal value of ROS was reduced to ~0.2% under physiological conditions [44,45]. Superoxide, one of the typical ROS, is generated by the respiratory chain in mitochondria, leading to ATP production [46]. Superoxide is converted to hydrogen peroxide (H_2_O_2_) by two types of intracellular superoxide dismutase (SOD) under physiological conditions: Cu/Zn-SOD in the cytosol and Mn-SOD in the mitochondrial matrix. Subsequently, the produced H_2_O_2_ is catalyzed into water and molecular oxygen by catalase or GSH peroxidase (GPx). Catalase can react with H_2_O_2_ but not other hydroperoxides, whereas GPx can react with both H_2_O_2_ and other hydroperoxides. For H_2_O_2_ detoxication, the *Km* value of catalase is high, whereas that of GPx is low [47]. Therefore GPx, but not catalase, mainly compensates for the peroxide detoxication under physiological conditions in neurons [18].

In mammalian tissues, there are four types of selenium-containing GPx, one of which, GPx-1, is widely expressed in most brain areas and various cell types [48]. GPx-1 is the most abundant cytosolic GPx, and it functions as an important antioxidative enzyme to interact with fatty acid hydroperoxides as well as H_2_O_2_ in the brain [49,50]. Additional GPx isozymes have been also identified as gastrointestinal GPx (GPx-2), plasma GPx (GPx-3), and phospholipid hydroperoxide GPx (GPx-4) [49]. In contrast to other GPx isozymes, GPx-4 can interact with a wide range of phospholipid hydroperoxides as well as H_2_O_2_ in the brain [49,51]. In the processes of H_2_O_2_ or organic hydroperoxide detoxication by GPx, GSH is oxidized to GSH disulfide (GSSG) [52,53], which is then regenerated as GSH by the reaction with GSH reductase (GR) [54]. This reaction of GR with GSSG is regulated by nicotinamide adenine dinucleotide phosphate (NADPH), which is provided as a substrate for supplying electrons to GSSG by NADPH regenerating enzymes, such as glucose-6-phosphate dehydrogenase (G6PDH), 6-phosphogluconate dehydrogenase, NADP^+^-dependent isocitrate dehydrogenase, malic enzyme, and mitochondrial nicotinamide nucleotide transhydrogenase [24]. Although the reaction rate of GR is limited by the supply of NADPH, neuronal GR is sufficiently active to rapidly regenerate GSH from GSSG [55]. GSH-*S*-transferase (GST) is a family of enzymes that detoxify a variety of electrophilic xenobiotics with GSH-*S*-conjugation. GST metabolizes anticancer drugs, insecticides, herbicides, carcinogens, and by-products of oxidative stress. In mammalian species, there are seven classes of cytosolic GST isoforms; α, μ, π, σ, θ, ω, and ζ [56]. GSH, GSSG and GSH-*S*-conjugates are released to the extracellular space via MRPs [57,58]. After the cleavage of GSH and its conjugates into the γ-glutamyl moiety and CysGly by γGT, the γ-glutamyl moiety is degraded to the corresponding amino acid and 5-oxoproline by the reaction with γ-glutamyl cyclotransferase. 5-Oxoproline, also known as pyroglutamate, is then converted to glutamate by the reaction with 5-oxoprolinase (ATP-hydrolysing). GSH is metabolized to the related amino acids, which are reused for GSH synthesis to form the γ-glutamyl cycle (Figure 1).

### 4.2. Oxidative Stress

Despite these GSH-related antioxidant systems, the brain is especially vulnerable to oxidative stress because of the relatively lower antioxidant enzymatic activities (SOD, GPx, GR, and catalase) compared to those in other tissues [59]; the brain requires a large amount of O_2_, leading to a high production of ROS and lipid peroxidation. Nitric oxide (NO) is an important molecule that regulates physiological cell function and signaling and also a free radical leading to the generation of reactive nitrogen species (RNS) under pathological conditions. NO is produced by NO synthase (NOS) activation, and its brain concentrations under pathological conditions are approximately 100-fold higher than those under normal conditions [60,61]. Neither superoxide nor NO is toxic unless they react non-enzymatically with each other to form peroxynitrite, which is a potent RNS in the brain [62]. Peroxynitrite is produced at an estimated rate of 50–100 μM per min with a half-life of ~10^−2^ s and can diffuse within one to two cell diameters (~5–20 μm) [63] to cause lipid peroxidation, antioxidant depletion, antioxidant enzyme inhibition, and DNA damage [62,63]. The rate of peroxynitrite formation is accelerated in a synergistic manner if both superoxide and NO productions are elevated under pathological conditions [62].

Although SOD can remove superoxide to prevent peroxynitrite formation, the rate of reaction between superoxide and NO is much faster than that between superoxide and SOD [64,65]. Moreover, peroxynitrite can inactivate Mn-SOD by the nitration of tyrosine residue [66]. GSH reacts non-enzymatically with ROS such as superoxide, NO, hydroxyl radical, and peroxynitrite [67]. H_2_O_2_ is generated by the SOD-catalyzing reaction to superoxide in a cell. H_2_O_2_ itself is not particularly toxic in the physiological range; however, H_2_O_2_ should be removed by catalase or GPx to prevent the subsequent formation of hydroxyl radicals, which is considered a potent oxidant targeting sugars, amino acids, phospholipids, DNA bases, and organic acids [68]. Hydroxyl radicals are a highly toxic ROS produced by peroxynitrite decomposition or the Fenton reaction, leading to the ferrous iron-dependent decomposition of H_2_O_2_[62,63]. No known enzymatic defense has been reported against hydroxyl radicals. However, the toxicity of hydroxyl radicals is limited in the cell because of its short half-life (10^−9^ s) [69]. Hydroxyl radicals can diffuse only within a small distance, which is 10,000 times smaller than that of peroxynitrite, and moreover the rate of Fenton reaction is a million times slower than that of peroxynitrite formation [62,64].

### 4.3. *S*-Glutathionylation

All of the amino acid residues can be subject to posttranslational modifications such as oxidation or disulfide formation. Cysteine, which comprises up to 3% of the amino acids in human proteins [70], works as the most reactive nucleophilic residue in proteins. Oxidative/nitrosative damage can alter the redox state of the cell by reacting with thiol residues of redox-sensitive proteins. ROS/RNS induce irreversible protein modifications, such as carbonylation or nitration, leading to permanent loss of the functions of proteins as enzymes, receptors, and transporters [71]. Moreover, these irreversible modifications lead the proteins to their misfolding or aggregation as the targets for degradation by the ubiquitin-proteasome system (UPS) [72].

GSH is the major thiol redox buffer to maintain intracellular redox homeostasis. Under oxidative stress conditions, GSH can lead to the reversible formation of mixed disulfides between protein thiol groups (*S*-glutathionylation) to prevent irreversible protein oxidation [73]. *S*-glutathionylation is a reversible modification for restoring these protein functions when the intracellular redox state returns to normal after the insults. Disulfide bonds are also reduced back to thiol residues by the reaction with disulfide reductases, such as thioredoxin or glutaredoxin [74], which facilitate normal protein folding [75]. Protein *S*-glutathionylation is an important adaptive cellular response to protect crucial protein functions in the cell.

### 4.4. Thiol Redox State

Although the intracellular GSH/GSSG ratio may vary, ranging from 10 to 300, the ratio is more than 100 under the steady state, while it can transiently shift to ~10 or less under oxidative stress conditions [76,77]. The cellular thiol redox state is usually expressed by the GSH/GSSG ratio, which regulates gene transcription [78]. DNA binding activity of the transcription factors, such as c-Jun, NF-κB, and Fos, are modulated by changes in the GSH/GSSG ratio [79–81]; the decreased ratio induces *S*-glutathionylation of the cysteine residue in the DNA binding domains, leading to the inhibition of DNA binding. The inhibition of GSH synthesis arrests the cell cycle in the S and G2 phases [82], whereas proliferating cells in the S and G2 phases of the cell cycle showed increased GSH levels in the nucleus [83]. These results indicate that GSH is required at the appropriate period for cell proliferation.

The cellular thiol redox state also regulates programmed cell death [84]. A decreased intracellular GSH/GSSG ratio induces anti-apoptotic protein Bcl-2 loss, cytochrome c release from mitochondria, and caspase activations by the induction of the p38 mitogen-activated protein kinase pathway, whereas an increased intracellular GSH/GSSG ratio prevents the programmed cell death [85].

## 5. Cysteine Uptake for Neuronal Glutathione Synthesis

### 5.1. The Excitatory Amino Acid Transporters

Mature neurons use extracellular cysteine, but not cystine, for their GSH synthesis [40,86,87]. Approximately 90% of total cysteine uptake in neurons is mediated by sodium-dependent systems, mainly the excitatory amino acid transporter (EAAT), also known as system XAG- [88,89]. EAATs are high-affinity sodium-dependent glutamate transporters that remove extracellular glutamate in the CNS [88,90]. EAATs form trimers, which *co*-transport anionic amino acid with three Na^+^ and one H^+^ while counter-transporting one K^+^[91,92]. This transport system can concentrate intracellular glutamate 5 × 10^6^-fold across the plasma membrane under equilibrium conditions [93]. To date, five EAATs have been cloned: glutamate aspartate transporter (GLAST, also termed EAAT1), glutamate transporter-1 (GLT-1, also termed EAAT2), excitatory amino acid carrier 1 (EAAC1, also termed EAAT3), EAAT4 and EAAT5 [88].

GLAST and GLT-1 are expressed mainly in astrocytes [88], and EAAC1, EAAT4 and EAAT5 are expressed in neurons [88]. EAAT4 and EAAT5 are exclusively localized to cerebellar Purkinje cells and the retina, respectively, whereas EAAC1 is widely expressed throughout the brain. Mature neurons *in vivo* do not express either GLAST or GLT-1, but do express EAAC1. GLAST and GLT-1 are expressed on the plasma membrane of glial processes surrounding the glutamatergic synapses, and EAAC1 is expressed mainly in the neuronal soma and dendrites, but not in the axons or synaptic terminals [88,94–97]. Indeed, the extracellular glutamate in the synaptic clefts is removed by glial EAATs, especially by GLT-1, but not by EAAC1 [97–99]. EAATs can transport not only extracellular glutamate but also cysteine into the cells [100]. In particular, EAAC1 can preferentially transport cysteine, rather than glutamate, into neurons. The relative efficacy of cysteine transport by EAAC1 is 10- to 20-fold higher than that of GLAST or GLT-1 [100]. These findings indicate that the main function of EAAC1 is not related to glutamatergic neurotransmission, but to cysteine metabolism in neurons.

### 5.2. Regulation of EAAC1 on Neuronal Glutathione Synthesis

**Figure 2:**
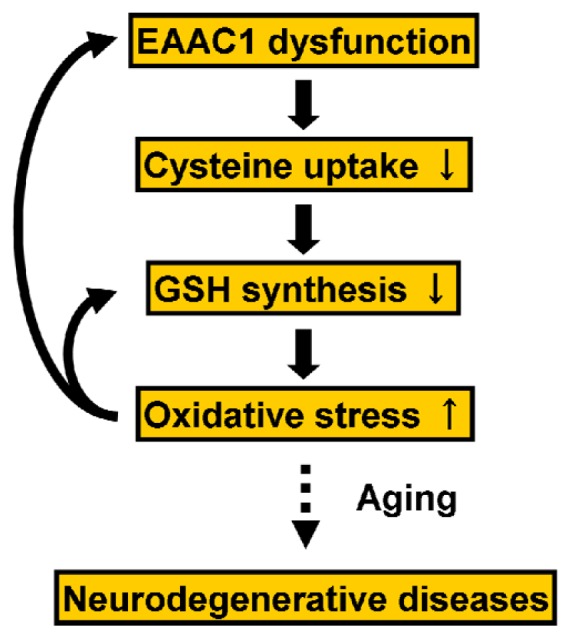
Possible mechanism of neurodegenerative diseases caused by GSH depletion via EAAC1 dysfunction.

Inhibition of EAAC1 expression by an antisense oligonucleotide significantly reduced the cysteine uptake, intracellular GSH levels, and cell viability against oxidative stress in cultured neurons [101]. Consistently, EAAC1-deficient mice show decreased GSH levels in the brain and vulnerability to oxidative stress. Interestingly, EAAC1-deficient mice also show brain atrophy, spatial learning and memory dysfunction, loss of dopaminergic neurons in the SN, and movement disorder at advanced ages but not when younger [102,103]. These findings suggest an involvement of EAAC1 dysfunction in brain GSH depletion leading to neurodegeneration (Figure 2).

The effect of EAAC1 on the glutamate/cysteine uptake into neurons is regulated by its expression on the plasma membrane. At steady state, only ~20% of total EAAC1 is expressed on the plasma membrane, whereas the cell surface expression was increased by twofold when stimulated by a protein kinase C activator, phorbol 12-myristate 13-acetate [104]. The cell surface expression of EAAC1 was also increased by stimulation with a phosphoinositide 3-kinase activator, platelet-derived growth factor [104]. EAAC1 translocation to the plasma membrane is negatively regulated by glutamate transport associated protein 3–18 (GTRAP3–18), which anchors EAAC1 in the endoplasmic reticulum [105]. GTRAP3–18 decreases the EAAC1-mediated cysteine uptake and GSH synthesis in neurons [11,106,107]. The precise regulatory mechanisms of EAAC1 and GTRAP3–18 are discussed in our previous reviews [108,109].

## 6. Inborn Errors in the GSH-Related Enzymes

### 6.1. γ-Glutamylcysteine Ligase

Disruption of the mouse *GCLC* gene causes embryonic lethality [110]. GCLM-deficient mice are viable and fertile with no obvious phenotype, but the GSH levels in their organs and plasma are low [111]. Loss of the *GCLM* gene induced premature senescence, increased intracellular ROS and DNA damage in primary fibroblasts [112]. Clinically, GCL deficiency is a very rare autosomal recessive disease that has been reported in only nine patients in seven families in the world. The patients have mutations in the gene encoding GCLC and show hemolytic anemia in all cases, and neurological symptoms such as spinocerebellar degeneration, mental retardation, peripheral neuropathy, myopathy and aminoaciduria in some cases [113]. The laboratory data show low GCL activity/levels and low GSH levels in erythrocytes and/or cultured skin fibroblasts. No promising treatment has been established.

### 6.2. Glutathione Synthetase

Disruption of the mouse *GS* gene also causes embryonic lethality [114]. Clinically, GS deficiency is the most common inborn error of GSH metabolism, reported in 77 patients in 65 families; it is characterized by autosomal recessive inheritance [114]. Some different mutations or epigenetic modifications of the human *GS* gene have been reported. The patients present hemolytic anemia, metabolic acidosis, and 5-oxoprolinuria. Severely affected patients also show progressive neurologic symptoms such as psychomotor/mental retardation, seizure, spasticity, ataxia, and intention tremor [113]. Laboratory data show increased γGluCys levels and cysteine in cultured fibroblasts and low GSH levels in erythrocytes and cultured fibroblasts. About 25% of the patients with GS deficiency die in the first year of life. The early administration of vitamin C (100 mg/kg/day) and/or vitamin E (10 mg/kg/day) improves the long-term clinical outcome [113].

### 6.3. γ-Glutamyl Transpeptidase

Human γGT deficiency is a very rare autosomal-recessive disease which has been reported in seven patients in five families worldwide [113]. The patients display increased GSH levels in plasma and urine, and in some cases, CNS involvement. Leukocytes or cultured fibroblasts from the patients exhibit low γGT activity. No mutation has been found in the patients. Clinically, no specific treatment has been established.

### 6.4. 5-Oxoprolinase

Human 5-oxoprolinase deficiency is a very rare autosomal-recessive disease reported in eight patients worldwide [113]. The patients exhibit low activity of 5-oxoprolinase in leukocytes or cultured skin fibroblasts, and 5-oxoprolinuria. Clinical manifestations of the patients include mental retardation, microcephaly, microcytic anemia, hypoglycemia, enterocolitis and renal stones. Clinically, no specific treatment has been reported.

### 6.5. Membrane-Bound Dipeptidase

Membrane-bound dipeptidase hydrolyzes dipeptides such as CysGly, which is produced in the subsequent process of GSH degradation after the reaction with γGT. The dipeptidase also catalyzes the conversion of leukotriene D4 to E4. Human dipeptidase deficiency is an extremely rare autosomal-recessive disease; it has been reported in only one patient worldwide [113]. The patient showed mental retardation, mild motor impairment, and deafness. The biochemical findings consisted of increased urinary excretion of both CysGly and leukotriene D4, and low dipeptidase activity in cultured fibroblasts and/or erythrocytes. No treatment has been reported yet.

## 7. Disorders of GSH Metabolism in Neurodegenerative Disease

Clinically, inborn errors in the GSH-related enzymes are very rare whereas disorders in GSH metabolism are common in some neurodegenerative diseases showing GSH depletion and increased levels of oxidative stress in the CNS (Figure 2). A recent *in vivo* method using nuclear magnetic resonance (NMR) spectroscopy makes it possible to measure GSH levels in the living brain [115]. Clinical studies using this method have demonstrated GSH depletion in patients with neurological disorders as described below. It is considered plausible that GSH depletion precedes neurodegeneration [116,117]. Many *in vivo* studies have shown both a GSH decline and increased ROS/RNS levels with aging in the brain [62,118]. The concept that older cells have less ability to prevent and remove oxidative damage is called “the free radical theory of aging” [119]. Aging also influences GSH homeostasis [118]. GSH depletion enhances oxidative stress, leading to neuronal degeneration [120–122]. Oxidative stress is involved in both normal aging and age-related neurodegenerative diseases [123,124]. Indeed, some neurodegenerative diseases have shown disorders of GSH metabolism in the brain, as discussed below.

### 7.1. Alzheimer’s Disease (AD)

AD is the most common age-related neurodegenerative disease. It is characterized by progressive dementia occurring in middle-aged or older populations. A recent clinical study using NMR spectroscopy showed reduced GSH levels in the brains of AD patients compared to healthy subjects [125]. No difference has been found in the concentrations of vitamins such as ascorbate or α-tocopherol in the CNS between AD and controls [126,127], suggesting a selective disorder of GSH metabolism in AD pathogenesis. Decreases in GPx and GST activities were observed in AD [128,129]. Genetic polymorphisms in the *GPx-1* and *GST* genes were identified as positive risk factors for AD [130,131]. The *Apo E* gene has a genetic polymorphism coding three different protein isoforms; ɛ2, ɛ3, and ɛ4. *Apo E* ɛ4 has been known as a risk factor for AD [132]. Brain tissues from AD patients with the ɛ4 allele of *ApoE* show decreased GSH levels and GPx and catalase activities compared to those of AD patients homozygous for the ɛ3 allele [133]. Notably, the GSH levels in erythrocytes were reduced not only in AD, but also in mild cognitive impairment (MCI), which is considered the preclinical stage of AD [134]. MCI patients also showed decreased GSH/GSSG ratios and SOD and GST activities in the hippocampus compared to age-matched controls [135]. These results suggest that disorders of GSH metabolism occur before the onset of AD.

AD is pathologically characterized by depositions of amyloid β (Aβ) plaques and neurofibrillary tangles (NFTs) in the brain. In the form of soluble oligomers, Aβ is most toxic, causing oxidative stress that leads to NFT formation and neuronal death [136,137]. Soluble Aβ oligomers inhibited the EAAC1-mediated cysteine uptake, resulting in a decrease in GSH levels in cultured human neuronal cells [138]. Consistently, postmortem brain tissues from AD patients show aberrant EAAC1 accumulation in pyramidal neurons of the hippocampus [139] and decreased GSH/GSSG ratios with the progression of AD [140]. These findings support the notion of EAAC1 dysfunction in AD pathogenesis.

### 7.2. Parkinson’s Disease (PD)

PD is the second most common age-related neurodegenerative disease. PD is a progressive, late-onset disease characterized clinically by presenting “TRAP” signs (tremor, rigidity, akinesia, and postural instability) [141]. The pathological hallmarks of this disease are the dopaminergic neurodegeneration in the SN and eosinophilic neuronal inclusions, called Lewy bodies, composed mainly of α-synuclein [142]. Most PD cases are sporadic, and less than 10% of the patients have a positive family history [143]. Both genetic and environmental factors are considered important in the etiology of PD [144,145]. PD is also characterized by a selective loss of GSH in the SN, but not in other parts of the brain [146]. As with AD patients, no change has been found in the concentrations of ascorbate or α-tocopherol in the CNS between PD patients and controls [147,148]. These findings also suggest a disorder of GSH metabolism as an underlying cause of PD. Postmortem brain tissues from normal individuals with incidental Lewy bodies and neuronal cell loss in the SN—who would be considered presymptomatic PD subjects—showed decreasing GSH levels in the SN compared to those of age-matched controls without Lewy bodies [149]. GSH depletion is considered an early event in the progression of PD [116]. Oxidative stress accelerates α-synuclein aggregation, which would also be facilitated by GSSG [150]. A decreased GSH/GSSG ratio in the brain may accelerate oxidative stress and Lewy body formation in the brain of an individual with PD.

Approximately 10% of PD patients show inherited forms [151]. Mutations in the *α-synuclein* gene were found in autosomal-dominant PD. The expression of familial PD-linked mutant human α-synuclein (A53T) in transgenic mice leads to late-onset neurodegeneration with an abnormal aggregation of α-synuclein in neurons [152]. The expression of the mutant A53T form of α-synuclein in dopaminergic neuronal culture caused GSH depletion with mitochondrial dysfunction [153].

Loss of function mutations in *parkin* were found in autosomal-recessive juvenile PD patients [154]. Parkin protein works as an E3 ubiquitin ligase in the process of ubiquitination to conjugate ubiquitin with specific substrates, including α-synuclein, leading to degradation by the UPS. The active sites of parkin are regions that are cysteine-rich and thereby sensitive to oxidative modification, which alters protein solubility and E3 ligase activity leading to dysfunction of UPS [155–157]. The UPS activity in the midbrain of aged parkin-deficient mice was damaged by GSH depletion [158].

*DJ-1* is also one of the causative genes for autosomal-recessive juvenile PD. DJ-1 exhibits protein interaction as a redox-dependent molecular chaperone [159] and up-regulates GSH synthesis during oxidative stress [160]. Oxidation of a conserved cysteine residue (Cys106) in DJ-1 regulates its chaperone activity against α-synuclein [159]. However, further oxidation of DJ-1 leads to loss of the ability and thus causes α-synuclein aggregation [159]. Indeed, DJ-1 is oxidatively damaged in the brains of idiopathic PD patients [161].

Dopaminergic neurons express high levels of EAAC1 in human brain [103,162]. EAATs are vulnerable to oxidative stress, resulting in reduced uptake function [163]. DA neurons are more susceptible to EAAC1 dysfunction than non-DA neurons [164]. Our previous study showed that dopaminergic neurotoxins, such as 1-methyl-4-phenyl-1,2,3,6-tetrahydropyridine and 1-methyl-4-phenylpyridinium, damage EAAC1 to reduce the neuronal cysteine uptake, leading to GSH depletion [165]. It is plausible that oxidative stress induces EAAC1 dysfunction, causing GSH depletion in PD.

### 7.3. Amyotrophic Lateral Sclerosis (ALS)

ALS is a progressive paralytic disorder characterized by the selective loss of motor neurons in the spinal cord and motor cortex. GSH reduction in the spinal cord has been reported in a mouse model of ALS [166]. Clinically, GSH levels and the activities of GR and G6PDH in erythrocytes are reduced in ALS patients [167]. These changes correlate with the disease progression. The spinal cord tissues obtained postmortem from patients with ALS showed increased protein glutathionylation in the gray matter [168] and reduced GST mRNA expression [169] compared to age-matched controls. Loss of GLT-1 has been reported in the spinal cord and motor cortex of ALS patients [170,171]. The expression of EAAC1 was reported to be slightly decreased in ALS motor cortex, although not significantly so [170]. Numerous studies have reported the involvement of oxidative stress in the pathogenesis of ALS [172]. Disorders of GSH metabolism might be a key risk factor in ALS. Riluzole, a neuroprotective drug for ALS patients, inhibits neuronal glutamate release and enhances astroglial glutamate uptake in the CNS [173,174]. A recent study showed an additional effect of riluzole on glial GSH synthesis under oxidative stress [175].

### 7.4. Progressive Supranuclear Palsy (PSP)

PSP is another age-related neurodegenerative disease; it is characterized by early postural instability, parkinsonism and a vertical supranuclear gaze palsy. PSP patients were recently shown to have decreased GSH levels in the SN [176]. A lipid peroxidation product, 4-hydroxy-2-nonenal (HNE), leads to the formation of cross-linked GSH-related enzymes to impair the enzymatic activities [177,178]. In the CNS of PSP patients, GPx conjugates with HNE, leading to impairment of its enzymatic activity [179]. Although recent studies have provided evidence that oxidative stress is involved in PSP pathogenesis [180], the precise mechanisms of declining brain GSH are not fully understood.

### 7.5. Huntington’s Disease (HD)

HD is a progressive neurodegenerative disease characterized by choreic movements caused by basal ganglia disorders. In the plasma of HD patients, the lipid peroxidation levels are higher and the GSH levels are lower than those of age- and gender-matched controls [181]. HD is associated with the expansion of a *CAG* trinucleotide repeat (in excess of 38 repeats) on the gene coding “*huntingtin*” with autosomal-dominant inheritance. In an *in vitro* study, decreased levels of GSH with elevated ROS levels were found in primary neurons from a knock-in mouse model of HD (HD^140Q/140Q^) in which a human *huntingtin* gene with 140 *CAG* repeats was inserted [182]. That study also showed EAAC1 dysfunction in the mouse model, which impaired cysteine uptake leading to GSH depletion in the neurons.

### 7.6. Multiple Sclerosis (MS)

MS is a neurological disorder characterized by inflammatory-mediated demyelination in the CNS. Although it is still arguable whether MS is a neurodegenerative disease or not, increasing lines of evidence support neurodegeneration as the major cause of irreversible neurological disability in MS patients [183]. Neuronal degeneration is a prominent feature in the brain of MS patients accompanied by neuritic transaction, neuronal apoptosis, and reduced neuronal and synaptic density [184]. Clinical studies by NMR spectroscopy have revealed lower brain GSH levels in MS patients than in controls [185,186]. Oxidative stress plays a major role in the pathogenesis of MS [187], however, the precise mechanism of GSH depletion is still unclear.

## 8. A Potential Approach to Increase GSH Levels in the Brain

A therapeutic strategy to increase neuronal GSH levels in the brain is a potential treatment for GSH-related neurodegenerative diseases, as mentioned above. However, no therapeutic drugs are available for increasing brain GSH levels at present. Orally dosed GSH is rapidly degraded in the gut, and intravenously administered GSH is rapidly oxidized to GSSG in the blood with a half-life of 2–3 min [28,29,188,189]. The administration of crude GSH does not seem promising for the treatment of neurodegenerative diseases.

*N*-acetylcysteine (NAC) is useful for the treatment of acetaminophen-induced hepatotoxicity by increasing hepatic GSH production or by its direct effect as an antioxidant. The systemic administration of NAC can also increase neuronal GSH levels in the brain by penetrating the BBB and the plasma membrane, even though neurons lack cysteine transporters [102]. Based on the favorable results of NAC treatments in some neurodegenerative models, some clinical trials using NAC have been in process for the treatment of AD or PD in the U.S. (ClinicalTrials.gov identifier: NCT01320527, NCT01370954, NCT01427517, and NCT01470027).

Considering EAAC1-mediated cysteine uptake as the rate-limiting step for neuronal GSH synthesis, a compound facilitating EAAC1 function might be a potential strategy for the treatment of GSH-related neurodegenerative diseases. To date, there is no promising drug for clinical use to modulate EAAC1 function exogenously. Alternatively, the endogenous modulation of protein-protein interactions might be crucial for enhancing EAAC1 function. GTRAP3–18 would be a potential target leading to an increase in neuronal GSH levels in the brain, but the physiological and pathological roles of human GTRAP3–18 function should be elucidated before it is used clinically.

## 9. Conclusions

GSH has a variety of pivotal functions in cells. Genetic disorders of GSH-related enzymes are rare, but GSH depletion is present in the pathogenesis of most major neurodegenerative diseases. Considering recent basic and clinical studies indicating that GSH depletion precedes neurodegeneration, neuronal GSH depletion would be a primary cause of neurodegenerative diseases (Figure 2). The development of drugs that target neuronal GSH synthesis would be a promising approach as a therapeutic strategy for neurodegenerative diseases.
